# Supplementary material for: Characteristics of Bipolar Patients with Cognitive Impairment of Suspected Neurodegenerative Origin: A Multicenter Cohort
Source: J Pers Med. 2021 Nov 11;11(11):1183. doi: 10.3390/jpm11111183 (PMC8620397; doi:10.3390/jpm11111183)
Supplement: Supplementary file 1 [file jpm-11-01183-s001.zip › jpm-1431518-supplementary.pdf]

Supplementary Table S1. Parkinsonism and antipsychotics intake in patient with BD and CI-SNO depending on DAT-Scan results

	**Presence of Dopamine deficiency (n=12)**	**Absence of Dopamine deficiency (n=22)**
	Number (Proportion)	Number (Proportion)
Parkinsonism	8 (67)	16 (72.7)
Antipsychotics intake (current or history)	6 (50)	12 (54.5)

Supplementary Table S2. Hippocampal atrophy in patient with BD and CI-SNO depending on CSF AD biomarkers profile

	**A+ (n=13)**	**A- (n=27)**
	Number (Proportion)	Number (Proportion)
Scheltens 0	5 (38)	11 (40)
Scheltens 1	1 (8)	3 (11)
Scheltens 2	5 (38)	9 (33)
Scheltens 3	2 (16)	4 (15)
Scheltens 4	0	0
